# Integrated Transcriptomic and Metabolomic Analyses Reveal the Effects of Grafting on Special Metabolites of *Acanthopanax senticosus* Leaves

**DOI:** 10.3390/molecules28124877

**Published:** 2023-06-20

**Authors:** Qi Wang, Kedan Deng, Jun Ai, Yingping Wang, Yougui Wang, Yueying Ren, Nanqi Zhang

**Affiliations:** 1 College of Traditional Chinese Medicine, Jilin Agricultural University, Changchun 130118, China; wangqi@mails.jlau.edu.cn (Q.W.); wangyingping@jlau.edu.cn (Y.W.); wangyougui@mails.jlau.edu.cn (Y.W.); 2 State Local Joint Engineering Research Center of Ginseng Breeding and Application, Changchun 130118, China; 3 College of Traditional Chinese Medicine, Jilin Agricultural Science and Technology University, Jilin 132101, China; dengkedan@jlnykjxy.wecom.work; 4 College of Horticulture, Jilin Agricultural University, Changchun 130118, China; juna@jlau.edu.cn

**Keywords:** *Acanthopanax senticosus*, grafting, transcriptomic, metabolomics

## Abstract

*Acanthopanax senticosus* (*A. senticosus*) is a member of *Acanthopanax Miq.* and is used in traditional Chinese medicine, and it has been found that grafting technology can be used to alter plant metabolite composition and transcriptome characteristics. In this study, shoots of *A. senticosus* were grafted onto the rootstocks of the vigorous Acanthopanax sessiliflorus (*A. sessiliflorus*) to improve its varietal characteristics. In order to investigate the changes in metabolites and transcriptional patterns in grafted *A. senticosus* leaves (GSCL), fresh leaves were collected from 2-year-old grafted *A. senticosus* scions, while self-rooted seedling *A. senticosus* leaves (SCL) were used as controls to analyse the transcriptome and metabolome. Metabolic profiles and gene expression patterns were further identified and correlated in special metabolite target pathways. The content of chlorogenic acid and triterpenoids in the GSCL was higher than in the control, while the quercetin content was lower. All these metabolic changes were associated with changes in the expression pattern of transcripts. Our results revealed the transcriptome and metabolome characteristics of GSCL. This may help to improve leaf quality in *A. senticosus* cultivation, suggesting that it is feasible to improve the medicinal quality of GSCL through asexual propagation, but the long-term effects need further investigation. In conclusion, this dataset provides a useful resource for future studies on the effects of grafting on medicinal plants.

## 1. Introduction

*Acantopanax senticosus* (Rupr. Maxim.) Harms (*A. senticosus*) is a perennial woody plant of the genus *Acanthopanax* in the family Araliaceae and is a traditional Chinese medicinal plant. For a long time, the roots and stems of *A. senticosus* have been applied extensively as a tonic, heart tonic and sedative. Numerous chemical, pharmacological and clinical studies have demonstrated the immunomodulatory, anti-stress, anti-fatigue and antitumour effects of *A. senticosus*, as well as its application in treating cardiovascular and cerebrovascular diseases. *A. senticosus* leaves (SCL) are generally recognised for their mild medicinal properties and healing powers, as a local medicinal herb in the Heilongjiang Province and as a new resource food ingredient in the Jilin Province. They have broad-spectrum effects due to their various phytochemicals, which are effective in treating cardiovascular diseases and hypoglycemia and have effects of anti-ageing, antioxidation, antibacterial and anti-inflammatory, anti-cancer, etc. [1,2,3,4]. These health properties are due to the presence of various positive health-promoting components in the leaves, such as triterpenoids, phenylpropanoids, flavonoids, polysaccharides, lignans and other individual compounds such as chlorogenic acid, quercetin, syringin, and isofraxidin [5,6,7,8]. The metabolites syringin and isofraxidin are active medicinal ingredients extracted from *A. senticosus*, produced through the phenylpropanoid biosynthesis pathway and used in Chinese Pharmacopoeia to determine the quality of the traditional Chinese herb *A. senticosus*. Chlorogenic acid is the special active ingredient of SCL [9], a simple phenylpropanoid compound produced by the plant under aerobic respiration via the shikimic acid pathway. In traditional Chinese medicine, it is often used as a medicinal ingredient and a simple preparation to clear heat and detoxify the body [10] and has good functions such as antiviral, antitumour cell, antibacterial, antiallergic, and regulates the activity of cytochrome P450 ligase [11]. According to the survey, more flavonoids, such as quercetin and other flavonoid glycosides, were included in SCL, which can reach 37.25% [12]. Quercetin, as the main flavonoid component, has anti-inflammatory, antioxidant, anti-atherosclerotic and good neuroprotective effects [13,14]. In addition, triterpenoids are the main secondary metabolites with various biological activities in SCL, which often have various important medicinal values in clinical practice, such as antitumour, anti-inflammatory, antiviral, cholesterol-lowering and immune-enhancing [15], and play an important role in determining the quality of the leaves. Jin et al. [16] showed that triterpenoids isolated from SCL significantly counteracted the adenosine diphosphate-induced platelet aggregation effect. This suggests that the concentrations and ratios of these metabolites can directly affect the potency and pharmacological effects of the leaves of *A. senticosus* and even the economic value of the herb.

In particular, grafted species differ in the composition and accumulation of metabolites when compared to ungrafted species, as influenced by the grafted rootstock and scion species [17,18,19,20]. For example, when peppers were grafted onto different rootstocks, CVS. Weishi (WS) and Buyeding (BYD), the accumulation of salicylic acid, benzoic acid, vanillin, lignin and polyamines in grafted peppers was increased to different degrees [21]. The accumulation of lycopene in grafted watermelon fruit was significantly increased when watermelon plants of the cultivar Ingrid were grafted onto the commercial hybrid rootstock PS 1313 [22]. Zhou et al. [23] found that tea tree scions ‘Yungui’ and ‘Fuyun NO.6’ grafted onto the rootstock ‘Duanjie baihao’ also differed in the relative contents of various substances such as flavonoids, organic acids and phenolic acids. This shows that the effect of grafting is not only related to the process, and the species used are strictly relevant. However, in some cases, the quality of the grafted plants may also be negatively affected by the grafting. However, this remains a controversial issue, as this decline in quality is not a universal phenomenon but is subject to specific scion-rootstock interactions, as well as specific combinations of growth conditions. At present, there is a complete lack of such information and no published studies on the effect of grafting on secondary metabolites of SCL, nor are there published data on *Acanthopanax sessiliflorus* (Rupr. Maxim.) Seem (*A. sessiliflorus*) rootstocks’ effect of SCL on secondary metabolites. Moreover, alterations in metabolites may also be associated with the RNA and proteins responsible for biosynthesis. As an example, transcriptional variations associated with secondary metabolism were elicited among grafted grapevine plants [24]. Therefore, *Acantopanax sessiliflorus* was selected as a rootstock, which has stronger and more vigorous growth compared with *A. senticosus*, to study the effect of grafting on the secondary metabolites of SCL to determine the mechanism of secondary metabolite accumulation and biosynthesis. This study will theoretically explain whether the quality of the leaves of *A. senticosus* can be improved by grafting, whether lines that perform well in production can be grafted together as a combination of good growing conditions to increase the diversity of the product and provide theoretical support for its production practice. The results of this research will also elicit a theory for making full use of the grafting technique to produce leaves of *A. senticosus*.

## 2. Results

### 2.1. LC-MS/MS-Based Metabolomics Reveals Metabolite Changes in GSCL vs. SCL

**Figure 1:**
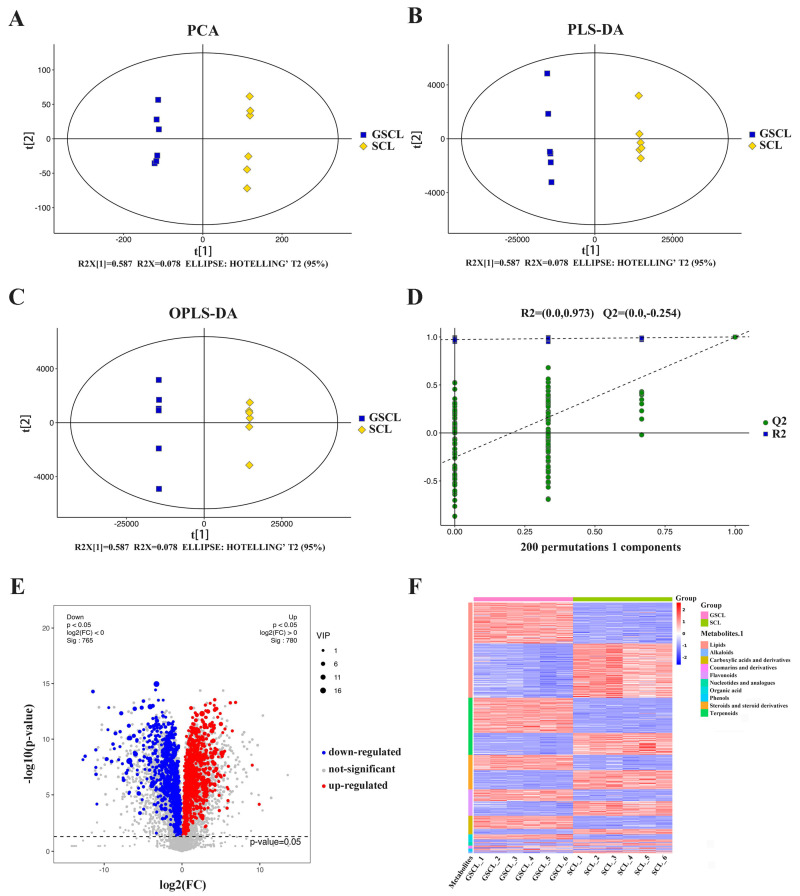
Multivariate statistical analysis of the differential metabolites based on LC-MS/MS. (**A**): Principal component analysis (PCA); (**B**): Partial least squares discriminant analysis (PLS-DA); (**C**): Orthogonal PLS-DA (OPLS-DA); (**D**): The 200-response sorting tests of the OPLS-DA model; (**E**): Volcano plot in GSCL vs. SCL; (**F**): Cluster heatmap of DMs classification in GSCL vs. SCL.

To investigate the metabolic differences in GSCL vs. SCL, LC-MS/MS was used to perform non-targeted metabolomic analysis. The basal peak plots of all samples showed that the instrument displayed a strong analytical signal, high peak capacity and good retention time reproducibility (Supplementary Figure S1). A total of 12,389 metabolites (6176 neg and 6213 pos) were identified. Based on these metabolites, PCA and (O)PLS-DA results showed significant differences between GSCL and SCL (Figure 1A–C), and the OPLS-DA model’s accuracy was illustrated through 7-fold cross-validation and 200 times RPT (Figure 1D). A total of 1545 differential metabolites (DMs) (780 upregulated and 765 downregulated in GSCL) were obtained by combining one-dimensional and multidimensional analyses to screen for DMs between the SCL and GSCL groups (Figure 1E). In order to show more clearly the changes in the relative contents of the more diverse DMs in GSCL vs. SCL, a cluster heat map of the differential metabolite classification was drawn using the software package R. The results revealed that the relative contents of most of the terpenoids, amino acids and their derivatives in the GSCL were significantly increased, while the relative contents of most of the metabolites in lipids, steroids and their derivatives, and flavonoids were significantly decreased (Figure 1F), indicating that grafting can change the types and contents of metabolites in SCL.

### 2.2. Special Differential Metabolite Analysis in GSCL vs. SCL

**Figure 2:**
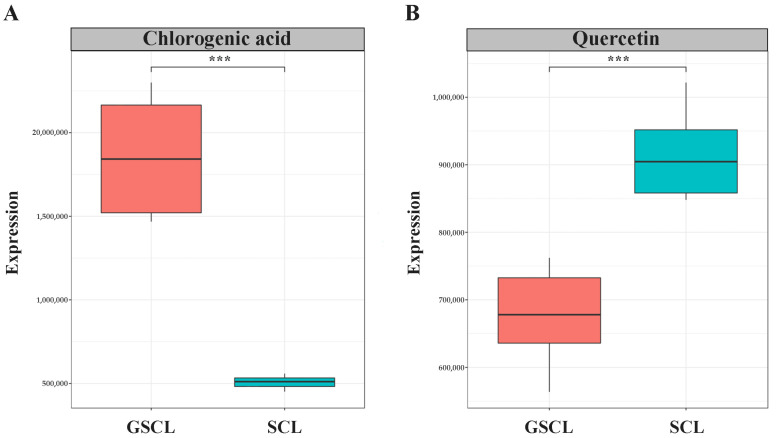
Box plot of the relative content of characteristic metabolites. (**A**): Relative content of Chlorogenic acid in GSCL vs. SCL; (**B**): Relative content of Quercetin in GSCL vs. SCL; ***: Indicates a significant difference between the two groups of treatments.

The relative quantification of peak areas of the same metabolite in different samples was utilised to analyse and compare the variation in the relative contents of the special metabolites syringin, isofraxidin, chlorogenic acid and quercetin of *Acanthopanax senticosus* leaves between different samples. The results showed that the special metabolites syringin and isofraxidin were obtained with a VIP < 1 in GSCL, indicating that their contribution to sample differentiation was small and not significantly different, while chlorogenic acid and quercetin were significantly different (Table 1). The relative content of chlorogenic acid was significantly higher, and the relative content of quercetin was significantly lower (Figure 2).

**Figure 3:**
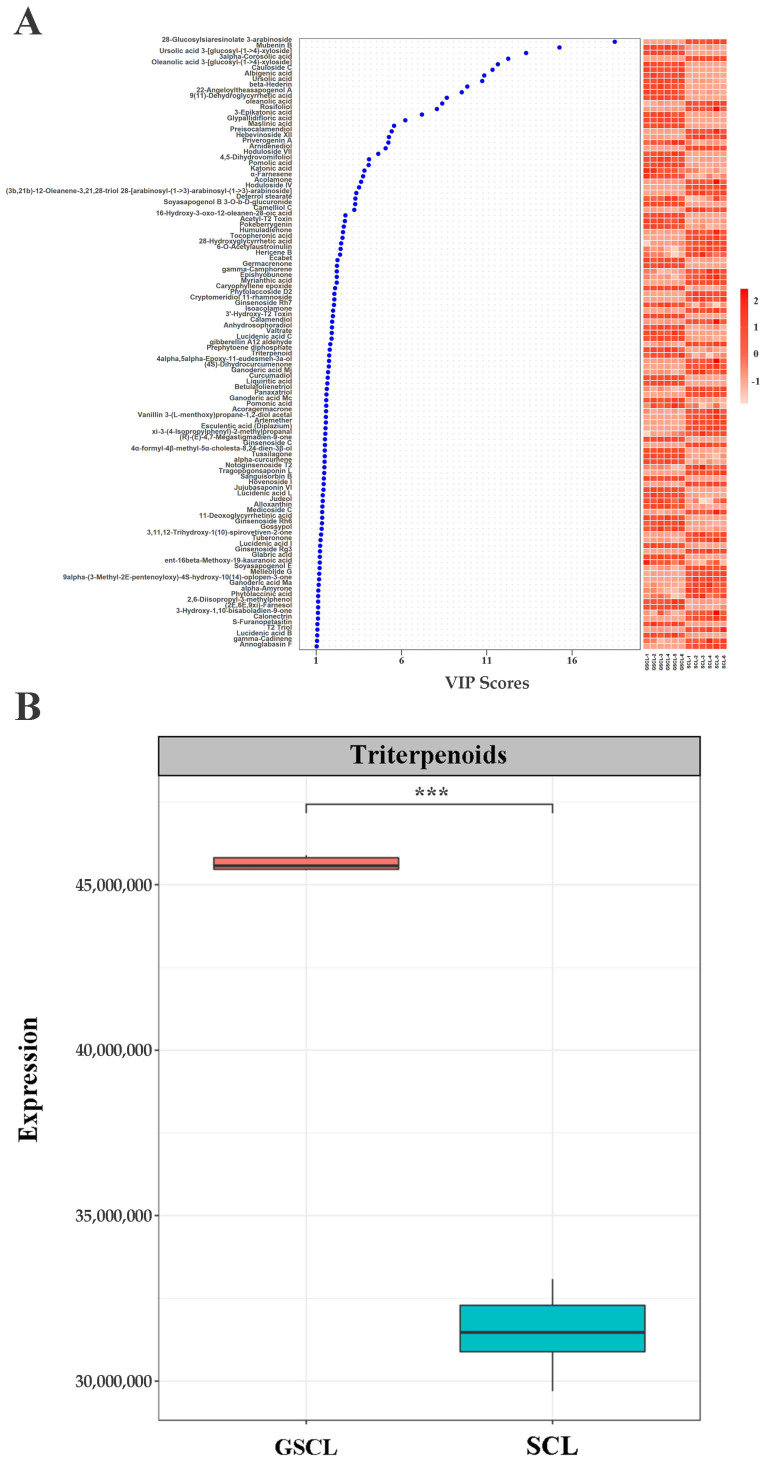
Metabolites with significant differences in annotation in GSCL vs. SCL. (**A**). The horizontal axis presents the samples, and the vertical axis presents the metabolites with a significant difference; (**B**). Boxplot of the relative content of triterpenoids. The horizontal coordinates are the leaf samples of *A. senticosus* before and after grafting, and the vertical coordinates are the relative expression of triterpenoids; ***: Indicates a significant difference between the two groups of treatments.

In addition, when screening differential terpene metabolites in GSCL vs. SCL, among the 1545 metabolites considered to be differentially accumulated, 109 were identified as terpenoids, including 7 monoterpenoids, 37 sesquiterpenoids, 6 diterpenoids, and 59 triterpenoids, most of which were elevated after grafting, with the highest proportion of triterpenoids, followed by sesquiterpenoids (Supplementary Table S1). By analysing the triterpenoids in GSCL vs. SCL and clustering them hierarchically according to their VIP ranking (Figure 3A), and quantifying them relatively in terms of the peak areas of triterpenoids in different samples, the results showed that the relative content of the special metabolites, triterpenoids, in GSCL increased significantly after grafting (Figure 3B). Given the relative content of these characteristic metabolites, it can be concluded that grafting affects the content of special metabolites of SCL, leading to quality differences in the samples before and after grafting.

### 2.3. De Novo Assembly, Analysis, and Functional Annotation of RNA-seq Data

For RNA-seq, equal amounts of high-quality total RNA from three biological replicates were mixed, purified and enriched to obtain mRNA, which was then reverse transcribed into cDNA and amplified by PCR to obtain four complementary deoxyribonucleic acid databases. High-throughput DNA sequencing using the Illumina HiSeq™ 2500 sequencing stage to sequence cDNA libraries yielded 41.81 Gb of clean data, averaging 6.97 Gb per sample. Over 95% of read quality scores were >Q30 (Supplementary Table S2). A total of 83,979 unigenes were produced by the trinity assembler, and their average length was 1065.7 bp. These results indicate that the data obtained meet the demands of the analysis that follows. To explore the possible functions of unigenes, Unigene was compared to the NR, SwissProt, KEGG, KOG, eggNOG and GO databases using diamond software, and functional analysis of Unigene was achieved by comparing Pfam databases via the HMMER software. The functional annotations gave unigenes of 49,941 (59.47%), 37,475 (44.62%), 11,848 (14.11%), 29,219 (34.79%), 46,513 (55.39%), 33,073 (39.38%) and 26,606 (31.68%), respectively, corresponding to the NR SwissProt, KEGG, KOG, eggNOG, GO and Pfam databases (Supplementary Table S3). The 64 GO terms associated with the 33665 annotated single genes were grouped into three classes, including biological processes (BP, 23), cellular components (CC, 20) and molecular functions (MF, 21). Within BP, CC and MF, the largest subcategories were ‘cellular processes’, ‘cellular parts’ and ‘binding’ (Supplementary Figure S2). Annotation results from mapping single gene annotations to KEGG showed that the assembled genes were annotated into 20 subcategories. Comparisons with other pathways revealed that more single genes were found in the ‘Translation’ (2072), ‘Carbohydrate metabolism’ (2066) and ‘Folding, sorting and degradation’ (1591) pathways (Supplementary Figure S3).

### 2.4. GO and KEGG Enrichment Analysis of Differential Expression Genes in GSCL vs. SCL

**Figure 4:**
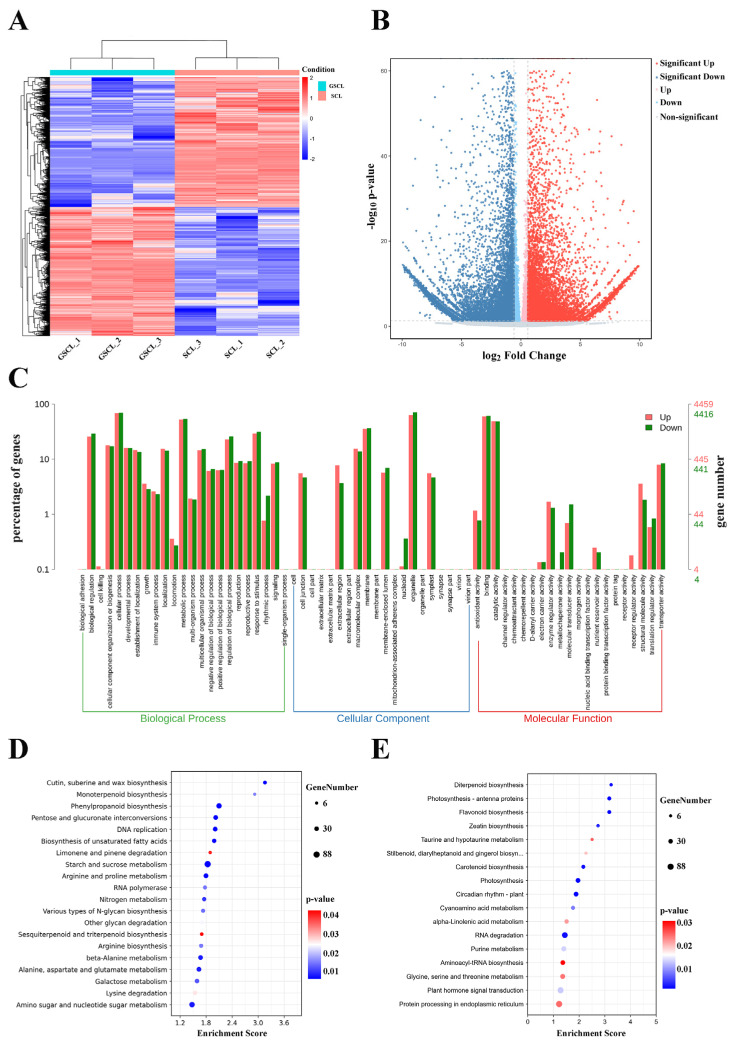
Functional classification of differentially expressed unigenes (DEGs). (**A**): Heat map about GSCL vs. SCL.Each of the samples is displayed on a column, and each gene is represented by a row. Blue indicates that the gene is expressed at a lower abundance in the tissue, while red indicates a higher abundance; (**B**): Volcano plot in GSCL vs. SCL. The red color indicates that the differential gene is upregulated in GSCL, while the blue color indicates that the differential gene is downregulated in GSCL; (**C**): DEGs ontology classification in GSCL vs. SCL at the GO level. Red means upregulated DEGs, and green means downregulated DEGs; KEGG enrichment analysis of upregulated (**D**) and downregulated (**E**) DEGs in GSCL. The enrichment score is the ratio between the number of DEGs in a pathway and all the annotated genes in this pathway. The larger the bubble, the more differential genes in the pathway.

Overall 20,198 differentially expressed genes (DEGs) (10,047 upregulated and 10,151 downregulated in GSCL) were identified between the GSCL and SCL samples (Figure 4A,B). Of these DEGs, 8875 single genes were annotated with GO terms. ‘Cellular process’ and ‘metabolic process’; ‘organelle’ and ‘membrane’ and ‘binding’ and ‘catalytic activity’ were the first two of BP, CC and MF, respectively (Figure 4C). KEGG enrichment analyses were performed to obtain insight into the synapse of specific metabolites, associated gene functions and gene interactions. The DEGs of those upregulated were enriched in phenylpropanoid biosynthesis, along with sesquiterpenoid and triterpenoid biosynthesis (Figure 4D). The DEGs of those downregulated were enriched in flavonoid biosynthesis (Figure 4E).

### 2.5. Differential Expression Genes in Special Metabolic Pathways in GSCL vs. SCL

**Figure 5:**
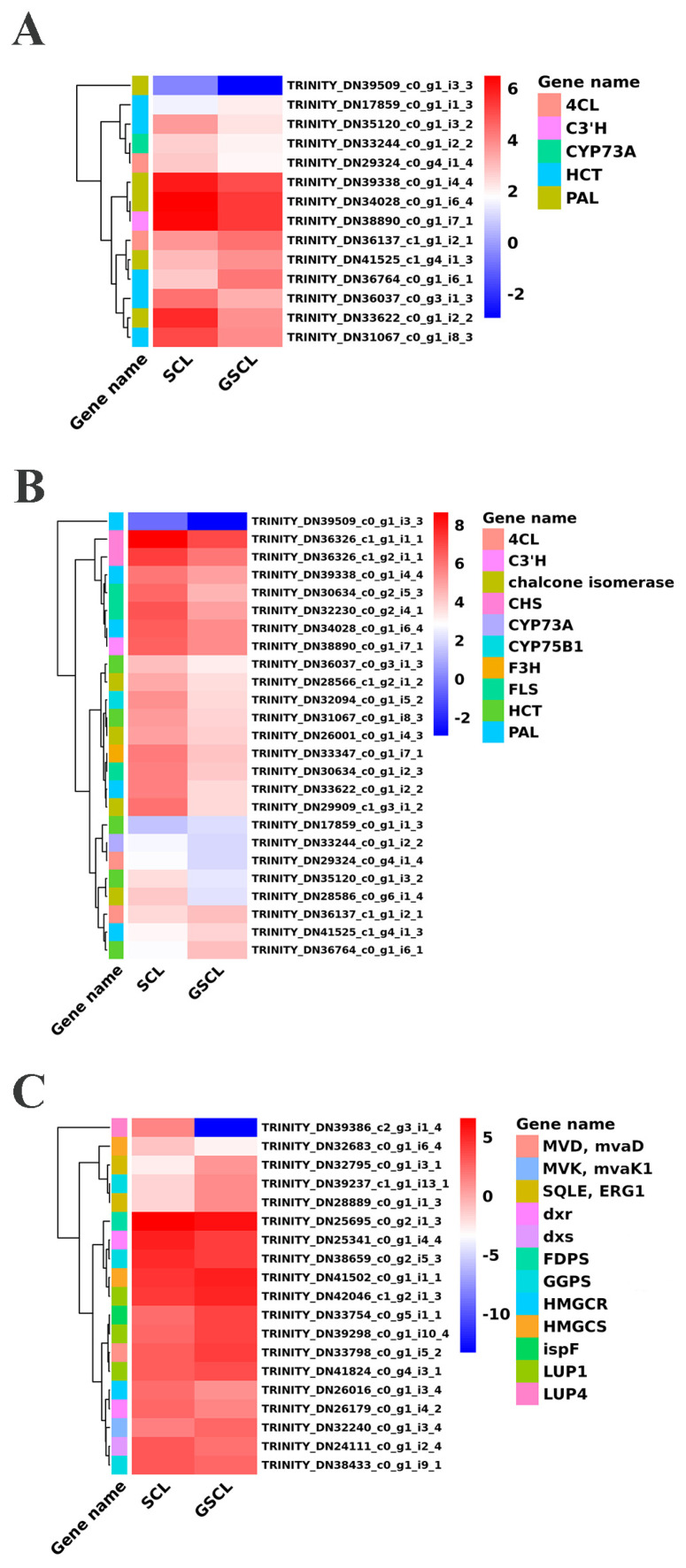
Heat map of the expression levels of DEGs in GSCL. (**A**): DEGs in chlorogenic acid biosynthesis pathway; (**B**): DEGs in quercetin biosynthesis pathway; (**C**): DEGs in terpenoid biosynthesis pathway. Each column and row represents a different sample and gene, respectively.

The above study revealed that the secondary metabolic pathways of GSCL are significantly different from those of SCL. DEGs regarding the special secondary metabolites chlorogenic acid biosynthesis, quercetin biosynthesis and triterpenoid biosynthesis were identified from the cDNAs of GSCL and SCL. A hierarchical clustering heat map was used to represent the expression of genes associated with the target pathway (Figure 5). Fourteen unigenes with significant differences in the chlorogenic acid metabolic pathway were identified, including the PAL gene (TRINITY_DN33622_c0_g1_i2_2, TRINITY_DN34028_c0_g1_i6_4, TRINITY_DN39338_c0_g1_i4_4, TRINITY_DN39509_c0_g1_i3_3, TRINITY_DN41525_c1_g4_i1_3), the CYP73A gene (TRINITY_DN33244_c0_g1_i2_2), the 4CL gene (TRINITY_DN29324_c0_g4_i1_4, TRINITY_DN36137_c1_g1_i2_1), the HCT gene (TRINITY_DN31067_c0_g1_i8_3, TRINITY_DN17859_c0_g1_i1_3, TRINITY_DN35120_c0_g1_i3_2, TRINITY_DN36037_c0_g3_i1_3, TRINITY_DN36764_c0_g1_i6_1) and the C3′H gene (TRINITY_DN38890_c0_g1_i7_1). In GSCL (relative to SCL), 10 unigenes were decreased, and 4 unigenes were significantly upregulated (Figure 5A). The results in the metabolic pathways involved in quercetin showed that a total of 25 significant DEGs were involved in the quercetin biosynthetic pathway in GSCL compared with SCL, of which 4 genes were upregulated, and 21 genes were downregulated, with the percentage of downregulated genes being 84%, and CHS, C3′H, CHS, F3H, FLS, CHI and CYP75B1 were reduced in the quercetin biosynthetic pathway (Figure 5B). In triterpenoid biosynthesis, 19 significantly different unigenes were identified, and 10 unigenes were significantly upregulated, including HMGCS (TRINITY_DN41502_c0_g1_i1_1), MVK/mvaK1 (TRINITY_DN32240_c0_g1_i3_4), MVD/mvaD (TRINITY_DN33798_c0_g1_i5_2), ispF (TRINITY_DN33754_c0_g5_i1_1), GGPS(TRINITY_DN39237_c1_g1_i13_1), SQLE/ERG1 (TRINITY_DN32795_c0_g1_i3_ 1, TRINITY_DN28889_c0_g1_i1_3) and LUP1 (TRINITY_DN39298_c0_g1_i10_4 and TRINITY_DN42046_c1_g2_i1_3, TRINITY_DN41824_c0_g4_i3_1) (Figure 5C).

### 2.6. Association Analysis Target to Special Metabolites

**Figure 6:**
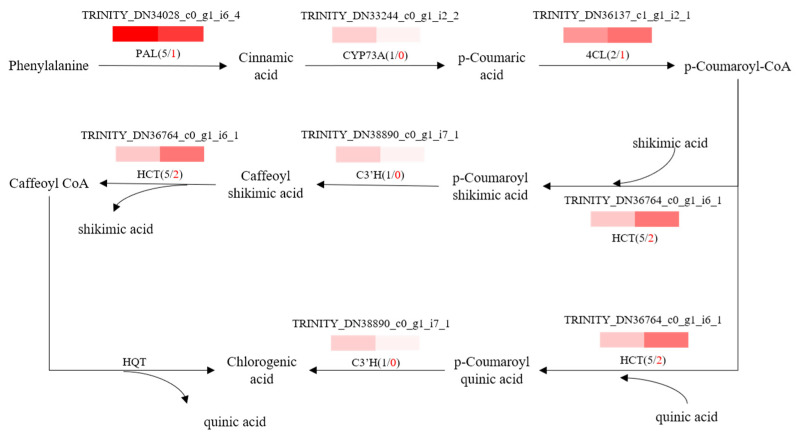
Correlation analysis of DEGs and DMs of the chlorogenic acid biosynthesis pathway in GSCL. The black number shows the total number of DEGs. The red number shows the upregulated number of DEGs in GSCL.

**Figure 7:**
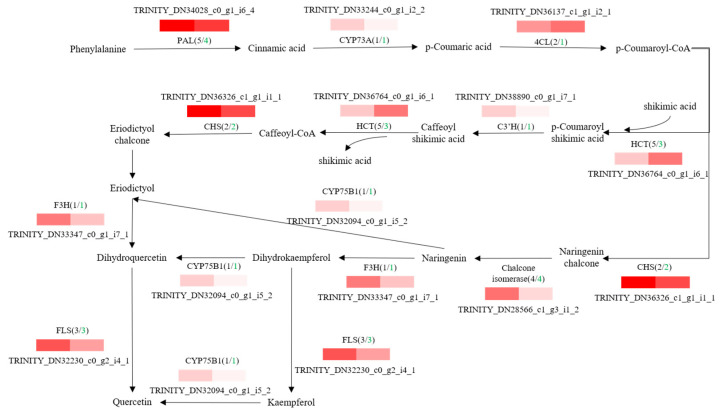
Correlation analysis of DEGs and DMs of quercetin biosynthesis pathway in GSCL. The black number shows the total number of DEGs, and the green number shows the downregulated number of DEGs in GSCL.

**Figure 8:**
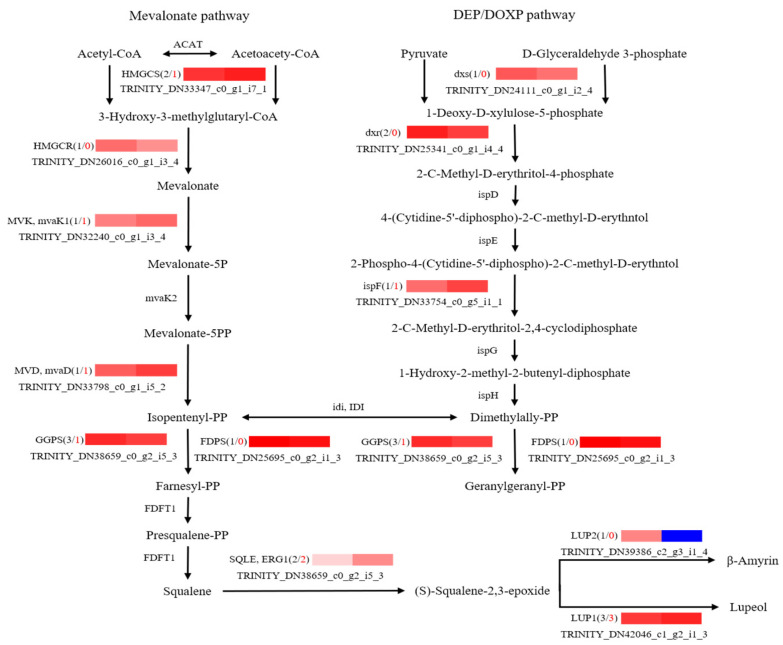
Correlation analysis of DEGs and DMs of triterpenes biosynthesis pathway in GSCL. The black number shows the total number of DEGs, and the red number shows the upregulated number of DEGs in GSCL.

To further explore potential regulatory mechanisms and to clearly show the relationship between DEGs and differential target metabolites in GSCL, a combined analysis of the biosynthetic pathways of chlorogenic acid, quercetin and triterpenoids was performed. The most highly expressed DEGs were shown to be in their metabolic pathway (Figure 6, Figure 7 and Figure 8). In the chlorogenic acid biosynthetic pathway, *p*-coumaric acid, caffeoyl shikimic acid and chlorogenic acid were noted to be significantly higher compared to SCL, while other compounds, such as cinnamic acid, were not reduced. The expression levels of genes of 4CL and HCT were all upregulated in GSCL, which increased chlorogenic acid biosynthesis in GSCL, indicating that grafting could alter the expression of genes related to the chlorogenic acid biosynthesis pathway in SCL (Figure 6). Compared with SCL, GSCL had a reduced content of kaempferol. Additionally, a total of 25 differentially expressed genes were involved in the quercetin biosynthetic pathway, and the percentage of downregulated genes was 84%. CHS, C3’H, F3H, FLS, CHI and CYP75B1, which were significantly related to the quercetin biosynthetic pathway, were all downregulated, indicating that quercetin biosynthesis-related genes are involved in regulating the biosynthesis of quercetin metabolites, suggesting that grafting has a regulatory effect on the expression of quercetin in *Acanthopanax senticosus* leaves (Figure 7). Critical gene expression patterns in triterpenoid biosynthesis are divided into two main steps, terpene backbone biosynthesis and triterpenoid biosynthesis. The terpene backbone biosynthesis pathway has two main pathways, Mevalonate and MEP/DOXP. Three differential genes involving the upstream Mevalonate pathway were expressed more in GSCL than SCL, one differential gene in the MEP/DOXP pathway was expressed more in GSCL than SCL, and two differential genes were expressed less in GSCL than SCL, indicating that it is the Mevalonate pathway that plays the major role. The expression of HMGCS, MVK/mvak1 and MVD/mvaD in the Mevalonate pathway was upregulated in GSCL, which was speculated to be possibly related to the high content of terpenoids in GSCL. In addition, ERG1/SQLE genes on the triterpene biosynthesis pathway also showed an upregulation trend in GSCL, suggesting that ERG1/SQLE may be a key gene for the synthesis of terpene skeletons into triterpene components in GSCL (Figure 8).

### 2.7. qRT-PCR Validation

**Figure 9:**
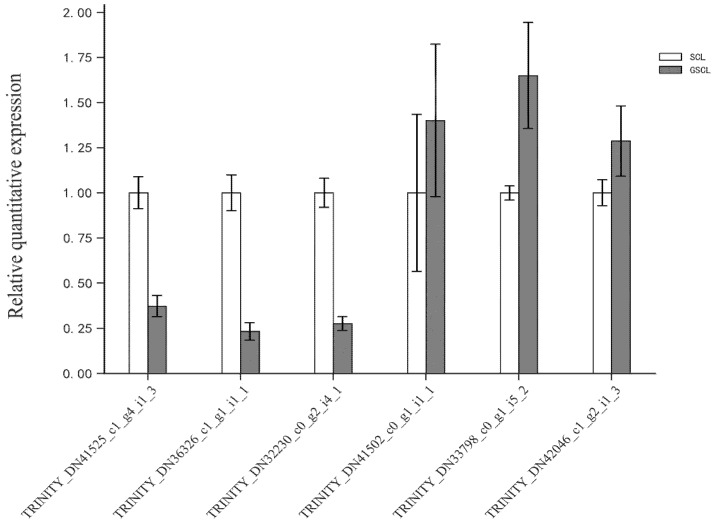
Validation of differentially expressed genes in GSCL vs. SCL by qRT-PCR.

In order to check the correctness and the reproducibility of the RNA-seq data, eight DEGs were selected for qRT-PCR analysis. The validation results were consistent with the trend of RNA-seq sequencing results, suggesting that the transcriptome analysis results were authentic and reliable (Figure 9).

## 3. Discussion

Metabolites, as products of the plant’s growth and development, usually accumulate in some of its specific tissues. It has been reported that the number of natural metabolites in plants may reach 200,000 [25]. These metabolites are important for the energy acquisition and health of plants and humans [26]. In the meantime, they are also a major source of clinical drugs and make a significant contribution to the agricultural sector and pharmaceutical industry. In this study, 1545 (780 upregulated and 765 downregulated) well-defined DMs were detected, and the special metabolites had an increased relative content of chlorogenic acid and triterpenoids and decreased relative content of quercetin. This makes them ideally suited for research on the mechanisms regulating the biosynthesis of metabolism because of the high diversity and variability of these plant metabolites and their ability to inform subsequent studies.

Moreover, the investigation strategy of histology provides new perspectives into systems biology, elucidating the relationship between related gene expression and metabolite accumulation. Plant cells can regulate their cellular metabolism to adapt to new conditions by initiating gene expression programs that respond to changes in conditions to facilitate their survival under different external conditions and by altering their own metabolites and thus exhibiting significant regulatory flexibility. In recent years, some scholars have begun to extensively investigate the potential mechanisms between grafted plant metabolites and the expression of enzyme activities or genes in related metabolic pathways. For example, some researchers have integrated transcriptomics and metabolomics to analyse grafted tea trees and oil teas to reveal important correlations between their specific secondary metabolite accumulation and genes [17,27]. In this study, to elucidate the role of grafting on the accumulation of metabolites in SCL and its regulatory mechanisms, an association analysis of metabolomics of specific metabolites with DEGs in the synthetic pathway was performed, showing the relationship between the expression levels of some genes and metabolites, indicating the effect of genes associated with the biosynthesis of these metabolites on the specific metabolites of GSCL. Chlorogenic acid, a phenylpropanoid produced by the shikimic acid pathway, is a condensed phenol produced by the combination of caffeic acid and quinic acid and is the main metabolite in GSCL. In this study, it was found that the high expression of 4CL and HCT in GSCL may have contributed to the synthesis of hydroxycinnamoyltransferase to generate *p*-coumaroyl quinic acid, which subsequently undergoes C3H hydroxylation to generate chlorogenic acid [28], which in turn increases the relative amount of chlorogenic acid. Studies on various plants have shown that HCT can promote chlorogenic acid synthesis [29,30,31]. Wen et al. [32] obtained two 4CL genes from the analysis of the chlorogenic acid metabolic pathway in pear fruits from the Xinjiang region. Therefore, it was suggested that grafting might further promote the synthesis of chlorogenic acid by promoting 4CL and HCT expression in GSCL.

In contrast, the relative content of the special metabolite quercetin was found to be reduced in GSCL, and the key enzymes of biosynthesis in the synthetic pathway (CHS, CHI, F3H, CYP75B1 and FLS) were downregulated, suggesting that these genes can regulate the differential synthesis of quercetin and reduce the biosynthetic activity of quercetin. The secondary metabolites downstream of the phenylpropanoid metabolic pathway are flavonoids and flavonoids, of which *p*-coumaroyl-CoA is a precursor of flavonoids and flavonoids [33,34], and CHS is the first key enzyme that leads the phenylpropanoid metabolic pathway into the flavonoid metabolic pathway, catalysing the transformation of *p*-coumaroyl-CoA into naringin chalcone [35] and driving the flow of upstream compounds in the phenylpropanoid synthetic pathway to the quercetin synthetic pathway [36,37], a key gene in quercetin biosynthesis, whose downregulation may lead to the blockage of quercetin biosynthesis. In addition, F3H can catalyse the formation of dihydrokaempferol from naringenin, further formation of kaempferol catalysed by FLS; and in the presence of CYP75B1, the 3′ and 5′ positions of the flavonoid B ring were hydroxylated to produce quercetin [36]. For example, the FLS-encoding flavonol synthase that was silenced in tobacco can result in a 17–53% reduction in quercetin content [38]. Xia et al. [39] compared the flavonoid metabolites of red ‘Summer Black’ (SB) and white ‘Shine Muscat’ (SM) grapes during the development of the fruit and found that the higher flavonol content of quercetin and kaempferol in SM was associated with a high expression of F3H and FLS. Quercetin can be synthesised from dihydroquercetin or kaempferol through FLS or CYP75B1, and some studies have reported higher quercetin content in the stem and leaves of Kurz. var. vinciflora (Kom.) L.T. Shen than its tuber due to the high expression of FLS, CYP75B1 and the aforementioned upstream genes [40]. Functional identification of CYP75B1 in Camellia sinensis suggested the role in the biosynthesis of flavonoids [41]. Therefore, this study suggested that grafting caused the downregulation of upstream genes and the crucial enzyme genes for quercetin biosynthesis, thereby decreasing the content of kaempferol and quercetin in GSCL.

Increased triterpenoid metabolites in GSCL may be associated with the upregulation of HMGCS, MVK/mvak1, MVD/mvaD, ERG1/SQLE and LUP1 expression. HMGCS is capable of catalysing the synthesis of 3-hydroxy-3-methylglutaryl-CoA by acetyl-CoA. It is a crucial rate-limiting enzyme for terpene backbone synthesis, the precursors of triterpenes in the MVA pathway, and has a positive regulatory effect on terpenoid synthesis [42,43,44]. MVK/mvak1 is the first of three sequential ATP-dependent enzymes in the mevalonate pathway for terpenoid synthesis, catalysing the production of mevalonate-5P from mevalonate. The isopentenyl-PP generated by the MVD/mvaD-catalysed decarboxylation of mevalonate pyrophosphate is a precursor material for the synthesis of terpenoids [45]. Thus, high expression of MVK/mvak1 and MVD/mvaD in GSCL promotes terpenoid backbone biosynthesis. In addition, two enzymes, ERG1/SQLE and LUP1, in the triterpene synthesis pathway, were significantly upregulated in GSCL. ERG1/SQLE cyclises squalene to (s)-squalene-2,3-epoxide and is a crucial enzyme in triterpenes synthesis [46]. LUP1, a lupinol synthase, is the main enzyme regulating lupinol synthesis in plants [47]. Its distribution as a triterpene is found in many plants, and its modification leads to various triterpenoids [48]. It is hypothesised that the synthesis of triterpenoids in GSCL is regulated by ERG1/SQLE enzymes and LUP1 enzymes, which further synthesise the terpene backbone into multiple triterpenoids.

## 4. Materials and Methods

### 4.1. Plant Materials

One-year-old seedlings of *Acantopanax senticosus* (Rupr. Maxim.) Harms (*A. senticosus*) and *Acanthopanax sessiliflorus* (Rupr. Maxim.) Seem (*A. sessiliflorus*) were purchased from Zhengyang Nursery (Liaoning, China). *A. senticosus* and *A. sessiliflorus* are each identical strains, each with stable genetic traits. All samples were identified by Prof. Jun Ai, College of Horticulture, Jilin Agricultural University, Changchun, China. The corresponding voucher specimens were stored in the State Local Joint Engineering Research Center of Ginseng Breeding and Application, China. We transplanted one-year-old *A. senticosus* and *A. sessiliflorus* in a nutrient bowl with organic substrates (Vloam: Vsand: Vpeatmoss = 3: 1: 1). The cleft grafting method [49,50] was carried out in March 2020, in the facility agricultural base of Jilin Agricultural University, China (latitude 43°48′ N, longitude 125°24′ E). In grafting experiments, one-year-old *A. senticosus* and *A. sessiliflorus* were used as scions and rootstocks, respectively, and the self-rooted seedlings of *A. senticosus* were controls. After grafting, all plants were transferred to a growth chamber where they were kept at a constant temperature of 20 ± 1 °C and a relative humidity of 80–90% for 2 weeks. Grafted plants were successfully transferred to a greenhouse for growth with the self-rooted seedlings of *A. senticosus* together. The test was divided into two combinations (grafted and ungrafted plants), and there were 60 plants in each combination. Each combination was replicated three times. Field management was handled according to conventional cultivation techniques, and all were managed by the same person. On 20 June 2022, three plants were randomly selected from each of the successful grafted and self-rooted seedlings, and three fresh leaves of the same leaf age were collected from each plant, for a total of 18 leaves. The graftings with *A. senticosus* as scion and *A. sessiliflorus* as rootstock were labeled as GSCL and the control self-rooted seedlings of *A. senticosus* were labeled as SCL. Samples were immediately frozen in liquid nitrogen and stored in a −80 °C refrigerator for metabolomics and transcriptomics analysis.

### 4.2. Sample Extraction and Metabolite Profiling

A total of 60 mg of sample was weighed and placed in a 1.5 mL centrifuge tube. Two small steel beads and a 600 μL mixture of methanol and water (V:V = 7:3, including internal standard l-2-chlorophenylalanine, 4 μg/mL) were spiked with each sample. The samples were pre-cooled at −40 °C for 2 min in a refrigerator and then milled for 2 min in a fully automated sample rapid grinder (Wonbio-E, Shanghai Wanbai Biotechnology Co., Ltd., Shanghai, China) with the grinding frequency set to 60 Hz. Samples were extracted using an ice-water bath for 30 min and left to stand overnight at −40 °C. Samples were centrifuged at low temperature for 10 min (13,000 rpm and 4 °C), and 150 µL of the supernatant was collected separately from each tube by syringe and filtered through an organic filter membrane (0.22 μm) and transferred to LC vials. The vials were kept at −80 °C until the LC-MS metabolomics analysis. All chemicals and solvents were of an analytical or LC/MS grade. All reagents used for extraction were pre-cooled in a −20 °C refrigerator prior to use. The analysis was carried out on an ACQUITY UPLC HSS T3 column (1.8 μm, 2.1 × 100 mm) in both positive and negative modes. Both contained 0.1% formic acid in water and acetonitrile as mobile phases, respectively. The gradient procedure is as follows: 0 min, 5% B; 2 min, 5% B; 4 min, 30% B; 8 min, 50% B; 10 min, 80% B; 14 min, 100% B; 15 min, 100% B; 15.1 min, 5% B and 16 min, 5% B. The column temperature was set at 45 °C, and the flow rate of the mobile phase was 0.35 mL/min. The injection volume was 2 μL. The mass spectrometry (MS) data were collected in bothESI+ and ESI- modes described by the following parameters: resolution (full scan) of 70,000; spray voltage of 3800 V in ESI+ (3000 V in ESI-); sheath gas flow rate of 35 arb; aux gas flow rate of 8 arb and a capillary temp of 320 °C. The mass spectra scan range was set as *m/z* 70–1000. A total of 12 samples (2 samples × 6 biological replicates) were used to observe the difference in metabolite composition between grafted plants and control leaves.

### 4.3. Multivariate Statistical Analysis and Metabolite Identification

Raw LC-MS/MS data were analysed using Progenesis QI V2.3 (Nonlinear, Dynamics, Newcastle, UK) software, such as basis line filtering, peak identifications, integration, retaining time correction, peak alignment and standardisation. Compounds were identified via The Human Metabolome Database (HMDB), Lipidmaps (v2.3) and METLIN databases, as well as the PMDB database, according to accurate mass numbers, secondary fragments, and isotopic distribution, qualitatively.

For the obtained data, ion peaks with > 50% missing values (0 value) within the group were deleted, and the 0 value was replaced by half of the minimum value. According to the fraction of the compound characterisation results, the compounds were screened, and those below 36 were considered to be inaccurate and were removed. The positive and negative ion data were combined into a data matrix for subsequent analysis.

The multivariate statistical analysis used principal component analysis (PCA) to visualise the general distribution between the samples and the stability of the entire analysis process. Partial least squares discriminant analysis (PLS-DA) and orthogonal partial least squares discriminant analysis (OPLS-DA) were used to distinguish the overall differences in metabolic profiles between the groups and to find the DMs between the groups. To prevent overfitting, 7-fold cross-validation and 200 response permutation testing (RPT) were used to assess the quality of the model. Further, based on the VIP values obtained in the OPLS-DA model, DMs with VIP values greater than 1.0 and *p*-values less than 0.05 were selected.

### 4.4. RNA Extraction, cDNA Library Construction and IIIumina Sequencing

Total RNA from GSCL and SCL was extracted using the mirVana miRNA Isolation Kit (Ambion, Naugatuck, CT, USA) plant RNA Kit based on the manufacturer’s instructions. The RNA integrity was assessed using an Agilent 2100 Bioanalyzer (Agilent Technologies, Santa Clara, CA, USA). Equal amounts of RNA from three biological replicates of each sample were used for cDNA library preparation.

Specific steps of transcriptome library construction and sequencing: According to the manufacturer’s instructions, the cDNA library was constructed using the A kit through a series of operations such as mRNA enrichment, fragment homogenisation, synthesis of first-strand cDNA and second-strand cDNA, purification and end repair of double-strand DNA, adding A tails and connecting sequencing connectors, fragment size selection and PCR amplification. After the constructed library passed the quality inspection, the prepared libraries were sequenced on the sequencing platform (HiSeqTM 2500, Illumina, San Diego, CA, USA).

### 4.5. De Novo Transcriptome Assembly and Functional Annotation

Transcriptome sequencing and analysis were performed by OE Biotechnology Co., Ltd. (Shanghai, China). Trimmomatic was used to process the raw data (raw read) [51] and rejected low-quality sequences for high-quality clean readings. The clean reads were spliced with the Trinity software (version: 2.4) paired-end splicing method to obtain the Transcript sequence [52]. The longest was selected as the unigene is based on sequence similarity and length. Unigene’s sequences were compared to the NCBI non-redundant (NR), Clusters of Orthologous Groups of proteins (COG/KOG) and Swissprot databases by using Blastx [53], and the threshold E-value was set to 10^−5^. The proteins with the highest sequence similarity to Unigene were used for functional annotation. According to the results of Swissprot, the Swissprot ID was mapped to the GO term to obtain the GO annotation of Unigene. Finally, Unigene was compared to the KEGG database to obtain the pathway annotation information [54].

### 4.6. Screening and Enrichment of Differentially Expressed Genes

Bowtie2 and eXpress software were used to analyse the FPKM and read counts value for Unigene [55,56,57]. DEGs were analysed by DESeq [58], and the screening criteria were false discovery rate (FDR) <0.01 and fold change ≥1.5. The GO and KEGG enrichment analysis of the differential gene was performed. Meanwhile, unsupervised hierarchical clustering was performed to discover the expression patterns of DEGs in different samples.

### 4.7. Quantitative Real-Time PCR Analysis

Reverse transcription was performed on RNA to be tested into cDNA using kit TransScript All-in-One First-Strand cDNA Synthesis SuperMIX for qPCR. After reverse transcription was complete, 90 μL Nuclease-free H_2_O was added, and it was stored in a −20 °C refrigerator. A total of six differential genes, PAL, CHS, FLS, HMGCS, MVD/mvaD and LUP1, were screened for qRT-PCR validation analysis in combination with metabolomic and transcriptomic analyses. All primers were synthesised by OE Biotechnology Ltd. (Nanjing, Jiangsu Province, China). The calculation of relative gene expression was performed via 2^−∆∆Ct^. GADPH was used as an internal reference gene. All reactions were repeated three times in the experiments. Gene names and primer numbers are listed in Supplementary Table S4.

## 5. Conclusions

In this study, 1545 DMs and 20198 DEGs were obtained by metabolomic and transcriptomic analyses, indicating that grafting can cause changes in metabolite recombination and transcript expression in SCL. Grafting was found to have the greatest effect on the special metabolites chlorogenic acid, quercetin and terpenoids in SCL in the study. Information on the differential expression patterns of genes and metabolites related to the leaves of *A. senticosus* before and after grafting was obtained by combined transcriptome and metabolome analysis. Both 4CL and HCT in the chlorogenic acid pathway are highly expressed in GSCL and promote chlorogenic acid biosynthesis. In contrast, six genes related to the quercetin synthesis pathway (CHS, C3H, F3H, FLS, CHI and CYP75B1), which are lowly expressed in GSCL, inhibited the accumulation of quercetin. Meanwhile, HMGCS, MVK/mvak1, MVD/mvaD and ERG1/SQLE in the terpene synthesis pathway were highly expressed in GSCL, promoting the accumulation of terpenoids, especially triterpenoids. The above results show the relationship between the effect of grafting on changes in special metabolites and gene expression patterns and reveal the transcriptional changes in secondary metabolites of medicinal plants via heterozygous grafting. This could have a positive impact with important implications for improving the production and breeding of *A. senticosus*.

## Figures and Tables

**Table 1:** VIP and *p*-values of special metabolites in GSCL vs. SCL.

Special Differential Metabolite	GSCL	SCL	VIP	*p*-Values
syringin	501,175.96 ± 87,306	546,293.52 ± 224,004	0.6022	0.6556
isofraxidin	11,210.14 ± 2332	17,301.89 ± 1466	0.5450	0.0003
chlorogenic	1,856,490.76 ± 385,710	507,624.08 ± 40,181	8.4377	<0.0001
quercetin	675,766.18 ± 74,694	914,886.84 ± 68,981	3.4413	0.0002

## Data Availability

The authors declare that all the data and plant materials will be available without restrictions. The original FASTQ files generated in the current study have been stored in the NCBI Sequence Read Archive (https://dataview.ncbi.nlm.nih.gov/object/PRJNA961992?reviewer=9j2qi29qk1oltccs261tmj3601, accessed on 4 June 2023).

## Supplementary Materials

The following supporting information can be downloaded at: https://www.mdpi.com/article/10.3390/molecules28124877/s1, Figure S1: Total ion chromatogram; Figure S2: GO analysis; Figure S3: KEGG annotation statistics; Table S1: Terpenoid metabolites with significant differences noted in GSCL vs. SCL; Table S2: Quality statistics of RNA-seq reads from GSCL and SCL; Table S3: Results of unigenes annotation from seven public databases; Table S4: Primers for qRT-PCR analysis.
