# Supplementary material for: Integrated Transcriptomic and Metabolomic Analyses Reveal the Effects of Grafting on Special Metabolites of Acanthopanax senticosus Leaves
Source: Molecules. 2023 Jun 20;28(12):4877. doi: 10.3390/molecules28124877 (PMC10301833; doi:10.3390/molecules28124877)
Supplement: Supplementary file 1 [file molecules-28-04877-s001.zip › molecules-2406601-supplementary.pdf]

Supplementary Material

Integrated Transcriptomic and Metabolomic Analyses Reveal the Effects of Grafting on Special Metabolites of *Acanthopanax senticosus* Leaves

Qi Wang [1,2], Kedan Deng [3], Jun Ai [4], Yingping Wang [1,2], Yougui Wang [1,2], Yueying Ren [1,2,*] and Nanqi Zhang [1,2,*]

1. Supplementary Figures

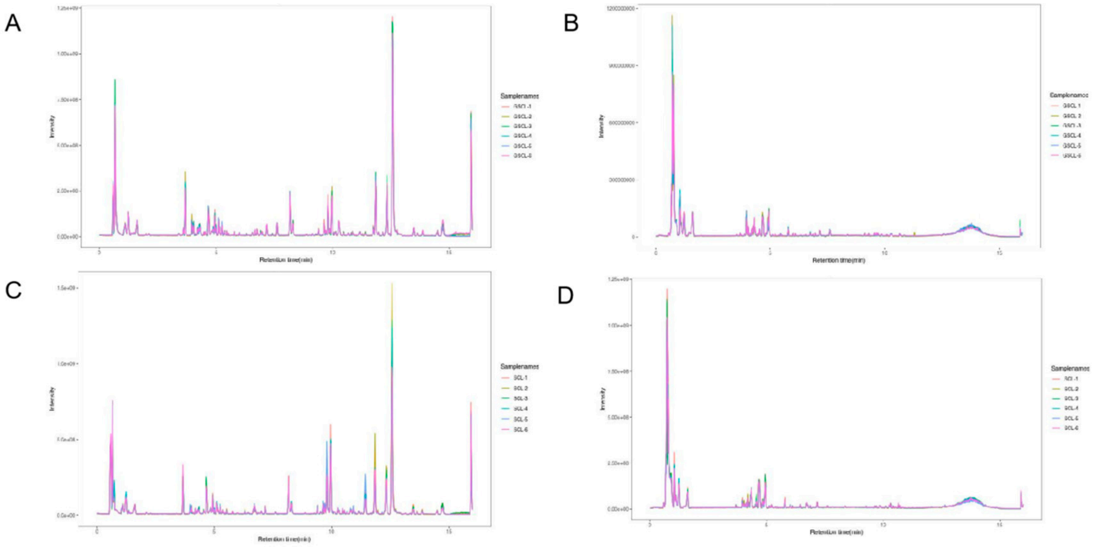

Supplementary Figure S1 Total ion chromatogram. A,B are the total ion chromatograms of GSCL in positive ion mode and negative ion mode, respectively. C,D are the total ion chromatograms of SCL in positive ion mode and negative ion mode, respectively. GSCL and SCL, pre- and post-grafting samples of *Acanthopanax senticosus* leaves, respectively, respectively. In each panel, each sample is displayed in a separate color and corresponds to 6 biological repeats (n = 6). The *x*-axis represents retention time, and the *y*-axis represents intensity.

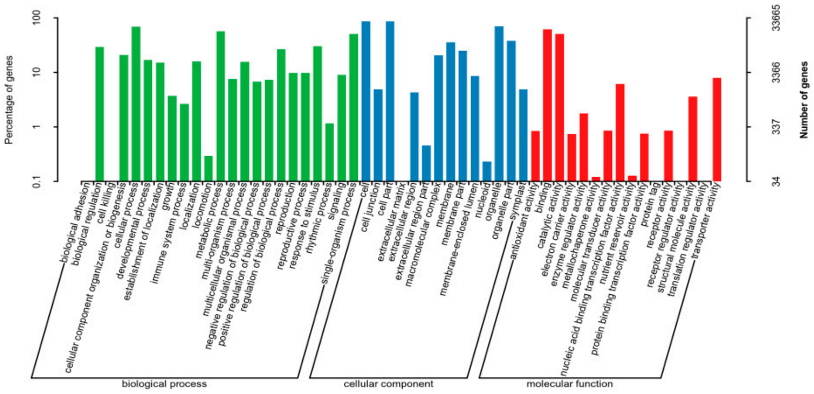

Supplementary Figure S2 GO analysis. The x-axis represents the GO functional classification, the y-axis on the left represents the percentage of genes annotated to this class, and the y-axis on the right

represents the number of unigenes.

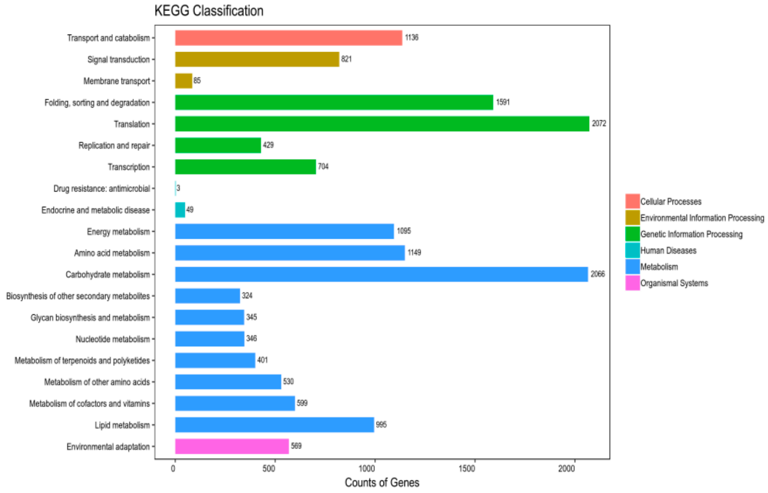

Supplementary Figure S3 KEGG annotation statistics. The x-axis represents the number of genes, the y-axis represents the name of the pathway, and the number on the right side of the column represents the number of genes annotated to the pathway.

2.Supplementary Tables

Supplementary Table S1 Terpenoid metabolites with significant differences noted in GSCL vs SCL.

NO.	Class	Compound name	GSCL	SCL	VIP	P-Value
1	Triterpenoids	28-Glucosylsiaresinolate 3-arabinoside	57331.3	623778	18.4	<0.0001
2	Triterpenoids	Mubenin B	8790365	442249	15.2	<0.0001
3	Triterpenoids	Ursolic acid 3-[glucosyl-(1->4)-xyloside]	3413443	262869	13.2	<0.0001
4	Triterpenoids	3alpha-Corosolic acid	167280.	283340	12.2	<0.0001
5	Triterpenoids	Oleanolic acid 3-[glucosyl-(1->4)-xyloside]	3045382	605528	11.6	<0.0001
6	Triterpenoids	Cauloside C	2435839	132002	11.3	<0.0001
7	Triterpenoids	Albigenic acid	2767999	650381	10.8	<0.0001
8	Triterpenoids	Ursolic acid	4953627	288636	10.7	<0.0001
9	Triterpenoids	beta-Hederin	1877517	130541	9.85	<0.0001
10	Triterpenoids	22-Angeloyltheasapogenol A	2820032	116036	9.52	<0.0001
11	Triterpenoids	9(11)-Dehydroglycyrrhetic acid	1430571	98266.	8.64	<0.0001
12	Triterpenoids	oleanolic acid	2069857	335092	8.38	<0.0001
13	Triterpenoids	3-Epikatonic acid	1845351	901028	7.19	<0.0001
14	Triterpenoids	Glypallidifloric acid	761359.	74635.	6.21	<0.0001
15	Triterpenoids	Maslinic acid	750959.	200679	5.56	<0.0001

NO.	Class	Compound name	GSCL	SCL	VIP	P-Value
16	Triterpenoids	Priverogenin A	2407518	184563	5.22	0.0003

cont. S1

NO.	Class	Compound name	GSCL	SCL	VIP	P-Value
17	Triterpenoids	Arnidenediol	36574.5	492416.	5.06	<0.0001
18	Triterpenoids	Hoduloside VII	434628.	40370.0	4.62	<0.0001
19	Triterpenoids	Pomolic acid	677984.	382619.	4.06	<0.0001
20	Triterpenoids	Katonic acid	910440.	588475.	3.81	0.0017
21	Triterpenoids	Hoduloside IV	5192.09	224427.	3.50	<0.0001
22	Triterpenoids	(3b,21b)-12-Oleanene-3,21,28-triol	32079.2	234660.	3.34	<0.0001
23	Triterpenoids	Camelliol C	53671.0	247827.	3.22	<0.0001
24	Triterpenoids	16-Hydroxy-3-oxo-12-oleanen-28-oic acid	137255.	7433.63	2.70	<0.0001
25	Triterpenoids	Pokeberrygenin	272935.	148975.	2.60	<0.0001
26	Triterpenoids	28-Hydroxyglycyrrhetic acid	396294.	511808.	2.45	<0.0001
27	Triterpenoids	Myrianthic acid	4412.79	90380.3	2.19	<0.0001
28	Triterpenoids	Phytolaccoside D2	6241.31	82409.0	2.06	<0.0001
29	Triterpenoids	Ginsenoside Rh7	270001.	188164.	2.03	<0.0001
30	Triterpenoids	Anhydrosophoradiol	89891.0	22860.6	1.91	<0.0001
31	Triterpenoids	Lucidenic acid C	130263.	66287.3	1.88	<0.0001
32	Triterpenoids	Prephytoene diphosphate	123790.	63072.3	1.78	<0.0001
33	Triterpenoids	Triterpenoid	531296.	469818.	1.75	0.0001
34	Triterpenoids	Ganoderic acid Mj	74851.8	132703.	1.69	0.0002
35	Triterpenoids	Liquiritic acid	60006.3	11832.3	1.64	<0.0001
36	Triterpenoids	Betulafolienetriol	61509.4	109346.	1.61	<0.0001
37	Triterpenoids	Panaxatriol	11277.5	56706.1	1.59	<0.0001
38	Triterpenoids	Ganoderic acid Mc	64532.5	19220.3	1.57	<0.0001
39	Triterpenoids	Pomonic acid	546625.	488056.	1.57	0.0048
40	Triterpenoids	Esculentic acid (Diplazium)	8587.00	51301.8	1.53	<0.0001
41	Triterpenoids	Ginsenoside C	0.00	40366.9	1.50	<0.0001
42	Triterpenoids	4α-formyl-4β-methyl-5α-cholesta-8,24-dien-3	43258.1	3507.26	1.49	<0.0001
43	Triterpenoids	Notoginsenoside T2	111791.	158178.	1.47	0.0009

44	Triterpenoids	Tragopogonsaponin L	12083.4	50907.1	1.46	<0.0001

cont. S1

NO.	Class	Compound name	GSCL	SCL	VIP	P-Value
45	Triterpenoids	Sanguisorbin B	42880.4	5969.65	1.43	<0.0001
46	Triterpenoids	Hovenoside I	27290.7	64515.9	1.43	<0.0001
47	Triterpenoids	Jujubasaponin VI	47807.0	11892.2	1.41	<0.0001
48	Triterpenoids	Lucidenic acid L	76411.7	42520.2	1.37	<0.0001
49	Triterpenoids	Medicoside C	146148.	180645.	1.34	<0.0001
50	Triterpenoids	11-Deoxoglycyrrhetinic acid	62881.8	30279.0	1.33	<0.0001
51	Triterpenoids	Ginsenoside Rh6	51432.5	18684.7	1.33	<0.0001
52	Triterpenoids	Lucidenic acid I	55896.0	28989.3	1.22	<0.0001
53	Triterpenoids	Ginsenoside Rg3	0.00	26624.2	1.21	<0.0001
54	Triterpenoids	Glabric acid	53766.1	28033.0	1.20	<0.0001
55	Triterpenoids	Soyasapogenol E	54850.3	80434.2	1.18	<0.0001
56	Triterpenoids	Ganoderic acid Ma	21154.2	46002.5	1.13	<0.0001
57	Triterpenoids	alpha-Amyrone	20454.1	43975.8	1.11	<0.0001
58	Triterpenoids	Phytolaccinic acid	71023.6	98764.1	1.10	0.0031
59	Triterpenoids	Lucidenic acid B	30817.9	10619.0	1.04	<0.0001
60	Sesquiterpenoid	Rosifoliol	381844.	169918	8.06	0.0002
61	Sesquiterpenoid	Preisocalamendiol	141125.	692459.	5.41	<0.0001
62	Sesquiterpenoid	Hebevinoside XII	378634.	894767.	5.25	<0.0001
63	Sesquiterpenoid	4,5-Dihydrovomifoliol	996957.	692232.	4.08	<0.0001
64	Sesquiterpenoid	α-Farnesene	431260.	167017.	3.73	<0.0001
65	Sesquiterpenoid	Acolamone	52748.9	317897.	3.62	0.0002
66	Sesquiterpenoid	Deterrol stearate	2410652	211628	3.28	0.0225
67	Sesquiterpenoid	Acetyl-T2 Toxin	316308.	183939.	2.67	<0.0001
68	Sesquiterpenoid	Humuladienone	254468.	380796.	2.56	<0.0001
69	Sesquiterpenoid	Tocopheronic acid	0.00	114953.	2.53	<0.0001
70	Sesquiterpenoid	Germacrenone	141089.	54568.6	2.20	<0.0001
71	Sesquiterpenoid	Epishyobunone	205621.	302913.	2.19	0.0001

72	Sesquiterpenoid	Caryophyllene epoxide	275489.	195985.	2.09	<0.0001

cont. S1

NO.	Class	Compound name	GSCL	SCL	VIP	P-Value
73	Sesquiterpenoid	Cryptomeridiol 11-rhamnoside	8512.03	87570.9	2.05	<0.0001
74	Sesquiterpenoid	Isoacolamone	89419.8	160720.	1.98	<0.0001
75	Sesquiterpenoid	3'-Hydroxy-T2 Toxin	78751.2	9828.16	1.96	<0.0001
76	Sesquiterpenoid	Calamendiol	119756.	194244.	1.92	0.0002
77	Sesquiterpenoid	4alpha,5alpha-Epoxy-11-eudesmen-3a-ol	7450.69	70411.8	1.74	0.0003
78	Sesquiterpenoid	(4S)-Dihydrocurcumenone	30246.2	85599.4	1.73	<0.0001
79	Sesquiterpenoid	Curcumadiol	75966.5	26685.6	1.66	<0.0001
80	Sesquiterpenoid	Acoragermacrone	65624.5	112458.	1.56	<0.0001
81	Sesquiterpenoid	Artemether	6574.04	50662.5	1.55	<0.0001
82	Sesquiterpenoid	(R)-(E)-4,7-Megastigmadien-9-one	89532.3	49237.4	1.50	<0.0001
83	Sesquiterpenoid	Tussilagone	203799.	163675.	1.48	<0.0001
84	Sesquiterpenoid	alpha-curcumene	124739.	84981.4	1.47	<0.0001
85	Sesquiterpenoid	Judeol	271520.	226483.	1.36	0.0063
86	Sesquiterpenoid	Gossypol	47825.0	16145.4	1.30	<0.0001
87	Sesquiterpenoid	3,11,12-Trihydroxy-1(10)-spirovetiven-2-one	3416.40	33798.4	1.27	<0.0001
88	Sesquiterpenoid	Tuberonone	20832.5	49854.4	1.24	<0.0001
89	Sesquiterpenoid	Melleolide G	14995.9	39770.4	1.17	<0.0001
90	Sesquiterpenoid	9alpha-(3-Methyl-2E-pentenoyloxy)-4S-hydroxy-10(	130.19	24666.1	1.15	<0.0001
91	Sesquiterpenoid	(2E,6E,9xi)-Farnesol	45857.3	24123.4	1.09	<0.0001
92	Sesquiterpenoid	3-Hydroxy-1,10-bisaboladien-9-one	77145.8	106402.	1.09	0.0077
93	Sesquiterpenoid	Calonectrin	2131.67	23872.9	1.09	<0.0001
94	Sesquiterpenoid	S-Furanopetasitin	25356.6	4539.41	1.06	<0.0001
95	Sesquiterpenoid	T2 Triol	14519.1	36383.4	1.05	<0.0001
96	Sesquiterpenoid	gamma-Cadinene	33249.7	56227.9	1.03	0.0007
97	Diterpenoids	6-O-Acetylaustroinulin	165836.	278264.	2.42	<0.0001
98	Diterpenoids	Ecabet	141442.	50179.2	2.24	<0.0001
99	Diterpenoids	gibberellin A12 aldehyde	62144.3	123493.	1.82	<0.0001

100	Diterpenoids	Alloxanthin	78026.7	44590.0	1.35	<0.0001

cont. S1

NO.	Class	Compound name	GSCL	SCL	VIP	P-Value
101	Diterpenoids	ent-16beta-Methoxy-19-kauranoic acid	86798.9	51690.7	1.19	0.0077
102	Diterpenoids	Annoglabasin F	2006.01	20725.8	1.01	<0.0001
103	Monoterpenoi	Soyasapogenol B 3-O-b-D-glucuronide	201787.	10296.6	3.28	<0.0001
104	Monoterpenoi	Hericene B	222493.	352109.	2.38	0.0029
105	Monoterpenoi	gamma-Camphorene	175729.	273905.	2.20	0.0002
106	Monoterpenoi	Valtrate	71546.2	3770.10	1.90	<0.0001
107	Monoterpenoi	Vanillin 3-(L-menthoxy)propane-1,2-diol	965.27	46236.5	1.55	<0.0001
108	Monoterpenoi	xi-3-(4-Isopropylphenyl)-2-methylpropanal	455527.	501203.	1.52	<0.0001
109	Monoterpenoi	2,6-Diisopropyl-3-methylphenol	46822.8	24028.4	1.10	<0.0001

Supplementary Table S2 Quality statistics of RNA-seq reads from GSCL and SCL.

Sample	RawReads	RawBases	CleanReads	CleanBases	ValidBases	Q30	GC
GSCL1	51.41M	7.71G	50.65M	7.21G	93.53%	95.20%	43.42
GSCL2	48.62M	7.29G	47.88M	6.91G	94.81%	95.04%	43.46
GSCL3	48.46M	7.27G	47.75M	6.89G	94.77%	95.01%	43.49
SCL1	50.67M	7.60G	49.92M	7.22G	95.03%	95.06%	43.54
SCL2	47.78M	7.17G	47.08M	6.80G	94.93%	95.12%	43.58
SCL3	48.00M	7.20G	47.35M	6.78G	94.11%	95.23%	43.55

Supplementary Table S3 Results of unigenes annotation from seven public databases

Database	Total unigenes	Annotated unigenes	Percent (%)	Non-annotated unigenes	Percent (%)
NR	83979	49941	59	34038	41
eggNOG	83979	46513	55	37466	45
Swiss-Prot	83979	37475	45	46504	55
KEGG	83979	11848	14	72131	86
KOG	83979	29219	35	54760	65
Pfam	83979	26606	32	57373	68
GO	83979	33073	39	50906	61
Total	83979	50461	60	33518	40

Supplementary Table S4 Primers for qRT-PCR analysis

Gene name	Gene ID	Primer	Sequence (5' - 3')	Product length
PAL	TRINITY_DN41525_c1_g4_i	Forward	GTGTGAAGGCTAGTAGTGAT	111
		Reverse	CACCTTGTTTAGTCCTCCTAT	111
CHS	TRINITY_DN36326_c1_g1_i	Forward	AATCCCAGAGAGCTGAGG	118
		Reverse	TGTTCGCTGTTGGTGATG	118
FLS	TRINITY_DN32230_c0_g2_i	Forward	TCCAAGTGCATGGACACA	99
		Reverse	GACCGGGACTTGAAGAAC	99
HMGCS	TRINITY_DN41502_c0_g1_i	Forward	GAACGGATCCTTGGCCAA	87
		Reverse	CATCTAATAAGACACTTGAG	87
MVD/mva	TRINITY_DN33798_c0_g1_i	Forward	GCTCGTGATCTTGAGGATGA	103
		Reverse	CAGCGGTAGGGAAATTGTT	103
LUP1	TRINITY_DN42046_c1_g2_i	Forward	ATGTGGAAGCTGAAGATTGC	84
		Reverse	GTATTGTCGTCCGACATGATT	84
